# Cochrane corner: topical anaesthetics for pain control following corneal abrasions

**DOI:** 10.1038/s41433-023-02762-y

**Published:** 2023-09-25

**Authors:** Annali L. Lawrenson, John G. Lawrenson

**Affiliations:** 1 Emergency Department, Epsom & St Helier University Hospitals NHS Trust, KT18 7EG Epsom, UK; 2 School of Health and Psychological Sciences, City, University of London, EC1V OHB London, UK

**Keywords:** Health care, Drug therapy

Traumatic corneal abrasions, causing a disruption of superficial corneal epithelial integrity, are a common presentation in both general and ophthalmic emergency departments [1–4]. Corneal epithelial defects can also occur following commonly performed ophthalmic surgical procedures, such as photorefractive keratectomy (PRK), alcohol delamination and corneal cross-linking. The high density of nociceptors in the cornea means that corneal abrasions are associated with significant pain, which is most intense at initial presentation and during the subsequent 24 to 48 h. The current NICE Clinical Knowledge Summary on corneal superficial injury [5], providing an evidence-based clinical guideline for UK primary care professionals, recommends simple oral analgesia such as paracetamol, with the addition of lubricating eye drops or ointment for additional pain relief. Whilst established dogma within ophthalmology advocates that topical anaesthetics should not be prescribed for corneal-abrasion-related pain due to safety concerns and the potential for abuse [6], this view has recently been challenged by emergency physicians [7, 8]. By contrast, surveys of eyecare professionals conducted in the USA reported almost universal opposition to this practice [9, 10]. The publication of the Cochrane Review by Sulewski et al. on ‘Topical ophthalmic anaesthetics for corneal abrasions’ is, therefore, very timely [11].

The review, which included nine randomised controlled trials (RCTs) recruiting 556 adult participants investigated the effectiveness and safety of self-administered topical anaesthetics for the relief of pain compared to a non-anaesthetic control group (placebo or other treatment). Four RCTs (314 participants) analysed individuals with traumatic abrasions presenting in an emergency department setting and five trials (242 participants) investigated post-surgical epithelial defects. Participants were supplied with one of the commonly used topical ophthalmic anaesthetics, specifically, tetracaine (used in 5 RCTs), proparacaine (3 trials) or lidocaine (1 RCT). Adjunctive oral analgesia was also used in all but one study, either using a designated dosing regime or ‘as required’. Pain intensity was evaluated using visual analogue scales (VAS) or numerical scales, where higher scores represented higher pain intensities. The effect of treatment on epithelial healing rates was also evaluated by slit-lamp biomicroscopy and the proportion of participants with adverse events was reported at the longest follow up time.

Topical anaesthetics had little or no effect in reducing self-reported pain for traumatic or surgically created abrasions from baseline to 24 h compared to an inactive control. However, based on low certainty evidence from one trial of participants with traumatic abrasions, anaesthetics may lower pain scores at 24–48 h. Only one post-surgical RCT reported on pain control up to 72 h and found that the intervention had little or no effect on post-surgical pain. Overall, for the trials included in the quantitative analysis, there was no significant reduction in resolution of epithelial defects between anaesthetic and control groups, nor in the proportion of eyes with complications, although the certainty of the evidence is very low for all safety outcomes mostly because of risk of bias and small sample sizes.

In recent years, Cochrane Eyes and Vision have produced a suite of reviews that have examined the management of corneal abrasions [12–14]. The findings of this latest review are unlikely to change practice and convince the eye care community of the safety of supplying topical anaesthetics for pain relief, even if provided in limited dose-vials and used for short periods. The review authors acknowledge that they had very low confidence in the efficacy data. Furthermore, the effect sizes for pain reduction were generally small and typically less than two units lower with anaesthetic on a 10-point VAS pain scale. Studies in emergency settings have attempted to define the minimum clinically important difference (MCID) for acute pain severity by measuring the change in VAS score associated with adequate pain control. A mean reduction in VAS of at least 30% was found to represent a clinically important difference in pain severity that corresponded to patients’ perception of adequate pain control. [15]

Although there were no safety concerns arising from the included trials, the review authors do not discount the potential for ocular morbidity, particularly in a ‘real world’ setting where patients are not as closely monitored as in the experimental studies. The growing opioid crisis in the USA has created an impetus for opioid-sparing pain management and this may explain the desire to seek alternative methods to control pain arising from corneal-abrasion associated pain in the emergency department. However, further research is still needed, with larger, better-quality trials and longer follow up times to provide the evidence base needed to justify a change in practice.
